# Exposure to preeclampsia *in utero* affects growth from birth to late childhood dependent on child’s sex and severity of exposure: Follow-up of a nested case-control study

**DOI:** 10.1371/journal.pone.0176627

**Published:** 2017-05-09

**Authors:** Kristine Kjer Byberg, Knut Øymar, Geir Egil Eide, Michele R. Forman, Pétur Benedikt Júlíusson

**Affiliations:** 1 Department of Pediatrics, Stavanger University Hospital, Stavanger, Norway; 2 Department of Clinical Science, University of Bergen, Bergen, Norway; 3 Centre for Clinical Research, Haukeland University Hospital, Bergen, Norway; 4 Department of Global Public Health and Primary Care, University of Bergen, Bergen, Norway; 5 Department of Nutritional Sciences, School of Human Ecology, University of Texas at Austin, Texas, United States of America; 6 Department of Pediatrics, Haukeland University Hospital, Bergen, Norway; Universidade de Sao Paulo, BRAZIL

## Abstract

**Background and objective:**

An adverse intrauterine environment may affect offspring growth and development. Our aim was to explore whether preeclampsia (PE) exposure *in utero* influences growth from birth to 13 years.

**Methods:**

In a nested case-control study, 229 children were exposed to PE (mild/moderate: *n* = 164, severe: *n* = 54) and 385 were unexposed. Length/height and weight were abstracted from records at birth, 3 and 6 months, 1 and 4 years, and measured along with waist circumference and skinfolds at follow-up at 11/12 (girls/boys) and 13 years (both sexes). Associations between PE and z-scores for growth were analyzed by multiple linear and fractional polynomial regression with adjustment for potential confounders.

**Results:**

In boys, exposure to mild/moderate PE was positively associated with linear growth after 0.5 years, but severe PE was negatively associated with linear growth in all ages. In girls, both exposure to mild/moderate and severe PE were negatively associated with linear growth. Exposure to PE was negatively associated with weight and body mass index (BMI) during infancy, but positively associated with weight and BMI thereafter, except that boys exposed to severe PE consistently had a lower weight and BMI compared to the unexposed. Exposure to severe PE only was positively associated with waist-to-height ratio at 11/12 (girls/boys) and 13 years (both sexes).

**Conclusions:**

From birth to adolescence, linear growth, weight and BMI trajectories differed between the sexes by severity of exposure to PE. In general, PE exposure was negatively associated with linear growth, while in girls; positive associations with weight and BMI were observed. This underlines fetal life as a particularly sensitive period affecting subsequent growth and this may have implications for targeted approaches for healthy growth and development.

## Introduction

Preeclampsia (PE) is diagnosed in 3–5% of pregnancies and may be a serious complication of the second half of pregnancy affecting both mother and child. It is characterized by maternal hypertension and proteinuria, and associated in its severe form with fetal growth restriction [1–3]. PE is classified by severity into mild, moderate and severe forms, with differences in pathophysiology, gestational age at diagnosis, fetal growth and outcomes [4].

According to the Barker hypothesis, a hostile intrauterine environment may be associated with low birth weight, with increased risk for having a shorter adult height, metabolic disorders, obesity, diabetes and cardiovascular diseases [5–7]. Children born small for gestational age frequently experience catch-up growth, although more catch-up in weight than in height [8].

PE has been associated with low birthweight, catch-up growth in infants and a high body mass index (BMI, kg/m^2^) during adolescence [9, 10]. Delay of thelarche but accelerated pubarche and increased risk for obesity in late childhood with subsequent metabolic anomalies and altered risk for cancer in adulthood, have also been reported in offspring exposed to PE compared to the unexposed [10–12].

The present study is a part of “The Stavanger study”, which has previously shown that exposure to PE is associated with a low birthweight, especially after severe PE [3], large waist circumference and a high BMI in girls at the onset of puberty [10]. However, there are limited data on longitudinal growth patterns of children exposed to PE from birth to late childhood. The aim of the present study was therefore to explore whether length/height, weight and BMI trajectories from birth to late childhood and waist circumference and skinfolds in late childhood vary by severity of PE and the child’s sex and differ in comparison with the unexposed. We specifically hypothesized that exposure to severe PE *in utero*, a known risk factor for small for gestational age, contributes to compromised linear growth and accelerated weight gain during childhood and that the pathways to growth may differ by severity of PE and by child’s sex.

## Methods

### Study population and design

From a population-based cohort including 12 804 deliveries during 1993–1995 at Stavanger University Hospital [3], the Medical Birth Registry of Norway was used to identify mothers with PE (n = 366) and controls (n = 659) to conduct a nested case control study: For each case, two matched controls were selected; one was the next delivery in the hospital (i.e. a birth date match) and one was the next born matched on maternal age (i.e. a risk factor for PE). The present study, “The Stavanger Study” described in detail elsewhere [10], was a follow-up of the nested case control study, aiming to study anthropometry, blood pressure and pubertal development in children after PE exposure *in utero*. The cases and controls were invited to participate in a first follow-up study. The ages at follow-up were selected to coincide with the age of pubertal onset (first follow-up) and menarche (second follow-up) of the children [10, 12]. Thus the mean age was 10.8 years (girls) and 11.8 years (boys) at the first follow-up, and at the second follow-up it was 12.8 years (both sexes), as shown previously [13]. As there were more missing participants in the controls than the case group, the original matching on maternal age and birth date was compromised, and maternal age was included as a potential confounder in the analyses. The analyzed sample included all children who participated in both follow-ups.

The study was approved by the Norwegian Data Inspectorate, the Regional Committee for Ethics in Medical Research Western Norway (Reference Numbers: First: 078–03, Second: 2010/1375) and the Institutional Review Boards of the National Cancer Institute (Reference Number: LAB09-0139) and University of Texas at Austin, United States (Reference Number: 2013-04-0036). At follow-up, participating mothers and children signed an informed consent/assent form.

### Exposures

PE was diagnosed based on blood pressure and proteinuria levels at gestational age (GA) 20 weeks on and further classified as mild, moderate or severe according to the Collaborative Low-dose Aspirin Study in Pregnancy (CLASP) criteria as specified previously [10, 14]. However, due to the pathophysiological similarity between mild and moderate PE, these two conditions were combined into one category for analyses [15].

### Outcomes

Birth length and weight were abstracted from hospital records for consenting participants.

In Norway, all children receive healthcare at well baby clinics with routine measurements of recumbent length (or standing height from 2 years of age) and weight from infancy to school age. At first follow-up, length/height and weight measurements from routine visits at well baby clinics at the target ages of 3, 6 and 12 months and 4 years were abstracted from clinical records. If a measurement was missing, the value from the closest visit in time was used and the exact age was recorded for all visits. Height, weight, triceps skinfold and waist circumference in offspring were measured twice each at both follow-ups, and subscapular skinfold at second follow-up, with the average used in the analyses. The measurements at follow-ups were performed by three specially trained nurses, as described previously [10]. Standard deviation scores (SDS) for height, weight, BMI, skinfold and waist measurements including the waist-to-height ratio, relative to sex and age, were calculated in R version 2.6.2 (R Development Core Team, Vienna, Austria). Calculating SDS according to WHO standards could put the data into an international perspective. However, growth of Norwegian children has been shown to deviate significantly from the WHO standards [16]. We have therefore used SDS based on the Norwegian growth reference in our calculations [17–19].

### Confounders

The potential confounders including categorical and continuous variables are presented below, and illustrated in a directed acyclic graph (S1 Fig):

*Child’s sex*: From medical records.

*Birth order* (recoded to firstborn or not): From maternal questionnaire at first follow-up.

*Maternal BMI*: Calculated from pre-pregnancy weight measurement at the first antenatal visit at primary healthcare examination during the first trimester of pregnancy and height measurement from first follow-up.

*Maternal smoking in pregnancy* (yes/no): Recorded at first antenatal visit.

*Maternal age at delivery*: Design variable.

*Maternal education at time of delivery* (< 9 years, 9–12 years, > 12 years): From maternal questionnaire at first follow-up.

GA and puberty staging were not adjusted for, as these are intermediate variables between the exposures and outcomes.

The questionnaires used have been shown as supporting information in a recent publication by Alsnes et al [20].

### Statistics

For descriptive statistics we used the mean and 95% confidence interval (CI) as well as median and lower and upper quartiles (Q_1_, Q_3_). For comparison between groups by severity of PE exposure Kruskal-Wallis one way analysis of variance and Mann-Whitney U-test were used for continuous variables that were not normally distributed, and compared by Gosset’s unpaired t-tests (Student, 1908) for approximately normally distributed variables.

Multiple linear regression analysis of growth (SDS for length/height, weight and BMI) over time,.i.e. at birth, 3 and 6 months, 1 and 4 years, and both follow-ups was computed using generalized estimating equations (GEE) taking into account correlations between repeated measurements in each child. To identify potential non-straight line effects of PE on growth, we used multiple fractional polynomial regression (MFPR) [21] adjusted for repeated measurements in each child by use of the mfpr and xtgee procedures in GEE of Stata 14. The effect of maternal PE (no, mild/moderate, and severe) on growth was studied using regression models with adjustment for potential confounders. Interactions with sex and age were tested using the likelihood ratio test. Finally, the identified fractional polynomials were plotted using R (version 3.2.1)

Also, associations between the severity of PE and SDS for skinfolds, waist circumference and waist-to-height ratio were analyzed by separate multiple linear regression analyses (general linear model: GLM) including the covariates above. A backward stepwise selection of confounders at P-values of < 0.05 was performed, with child’s sex and maternal smoking forced into the final model with adjustment for significant confounders.

The estimated coefficients (b), 95% CI and P-values from Wald’s chi-square-test (GEE) and the *F*-test (GLM) are reported. All tests were 2-tailed and P-values ≤ 0.05 were considered statistically significant.

STATA SE14 (StataCorp. 2015. *Stata Statistical Software*: *Release 14*. College Station, TX: StataCorp LP.) was used for the GEE analyses, and IBM SPSS for Windows (version 22.0.0, Chicago, Ill., USA) was used for the GLM analyses.

## Results

### Characteristics of the participants

A detailed description of the cohort is previously published [13]. Briefly, out of 366 exposed and 659 unexposed invited children, 229 (63%) of the exposed (mild/moderate: n = 164, severe: n = 54) and 385 (59%) of the unexposed children assented to the first follow-up. 182 (50%) of the exposed (mild/moderate: n = 127, severe: n = 46) and 286 (43%) of the unexposed children assented to the second follow-up. Information about PE severity was missing for 11 children at the first follow-up, and for 9 children at the second follow-up. Maternal age at delivery was greater in children who did than did not assent to the second follow-up. Otherwise, there were no significant differences in perinatal characteristics between children who did and did not assent to the first and the second follow-ups (Table 1) [13]. GA at birth differed between the three PE exposure groups (Kruskal-Wallis test: P < 0.001). Pairwise comparisons showed that children exposed to severe PE had a lower GA at birth than those exposed to mild/moderate PE and the unexposed (Mann-Whitney U-test for both comparisons: P < 0.001) and that children exposed to mild/moderate PE had a lower GA at birth than the unexposed (Mann-Whitney U-test: P < 0.001). Median weeks (Q_1_, Q_3_) were, respectively; 36.1 (32.0, 38.6), 39.1 (37.6, 40.1) and 40.1 (39.3, 41.0). BMI SDS at the first follow-up was higher in girls who only assented to first follow-up than in those who assented to both follow-ups (mean difference BMI: 0.44 kg/m^2^; 95% CI: 0.20 to 0.68; unpaired t-test P < 0.001).

**Table 1:** Comparison of invited children to the Stavanger Study (n = 1025) according to assenting status to follow-ups^a)^.

Variable	First follow-up^a)^							Second follow-up^a)^						
	Assented (n = 617)			Did not assent (n = 408)				Assented (n = 470)			Did not assent (n = 555)			
	n			n			p	n			n			p
Gender: boys, *n (%)*	613	293	(47.8)	408	220	(53.4)	0.056^b)^	468	230	(49.1)	553	283	(51.2)	0.530^b)^
Preeclampsia, *n (%)*	614	229	(37.3)	438	165	(37.7)	0.676^b)^	469	182	(38.8)	583	212	(36.4)	0.184^b)^
Maternal age, years, *mean*, *95% CI*	612	28.0	(27.7, 28.4)	408	27.6	(27.2, 28.1)	0.189^c)^	467	28.2	(27.8, 29.7)	553	27.6	(27.2, 28.0)	0.030^c)^
Gestational age, weeks; *median*, *Q*_*1*_, *Q*_*3*_^d)^	602	39.9	(38.6, 40.7)	372	39.9	(38.4, 40.9)	0.718^e)^	458	39.9	(38.6, 40.9)	516	39.9	(38.5, 40.7)	0.781^e)^
Birth weight SDS^f)^	601	-0.16	(-0.27, -0.08)	341	-0.07	(-0.19, 0.05)	0.185^c)^	457	-0.20	(-0.31, -0.09)	485	-0.08	(-0.18, 0.02)	0.123^c)^

*Abbreviations*: CI = confidence interval

^a)^ First follow-up at the ages of 10.8 years (girls) and 11.8 years (boys); Second follow-up at 12.8 years;

^b)^ Exact chi-square test;

^c)^ Gosset’s t-test;

^d)^ Q_1_, Q_3_ = Lower and upper quartiles;

^e)^ Mann-Whitney U Test;

^f)^ SDS = standard deviation score

### PE and outcomes

**Fig 1:**
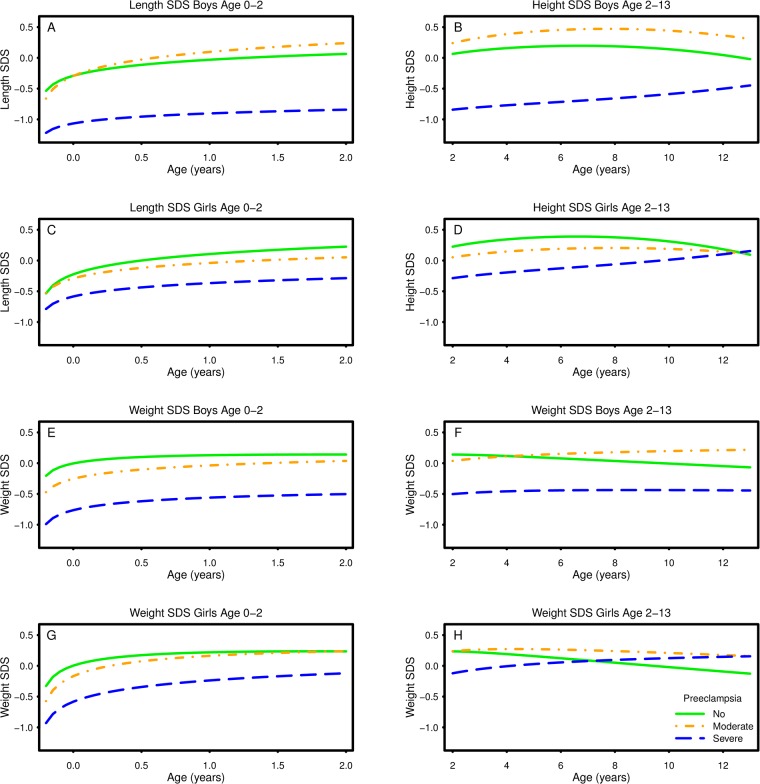
Plots of predicted length/height standard deviation score (SDS) (A-D) and weight SDS (E-H) vs. age according to sex and severity of preeclampsia. Key to figures: Solid line = Unexposed, Dash-dot line = mild/moderate preeclampsia, Dashed line = Severe preeclampsia. Each figure represents the fractional polynomial (FP) with the best fit for each measure (X), i.e. FP (0, 3) = b_1_ln(X) + b_2_X^3^; FP (0, 0.5) = b_1_ln(X) + b_2_√X. The plots are adjusted for sex, age, birth order, maternal age, smoking, BMI, education and an interaction between preeclampsia and age. Details appear in Table 2.

**Fig 2:**
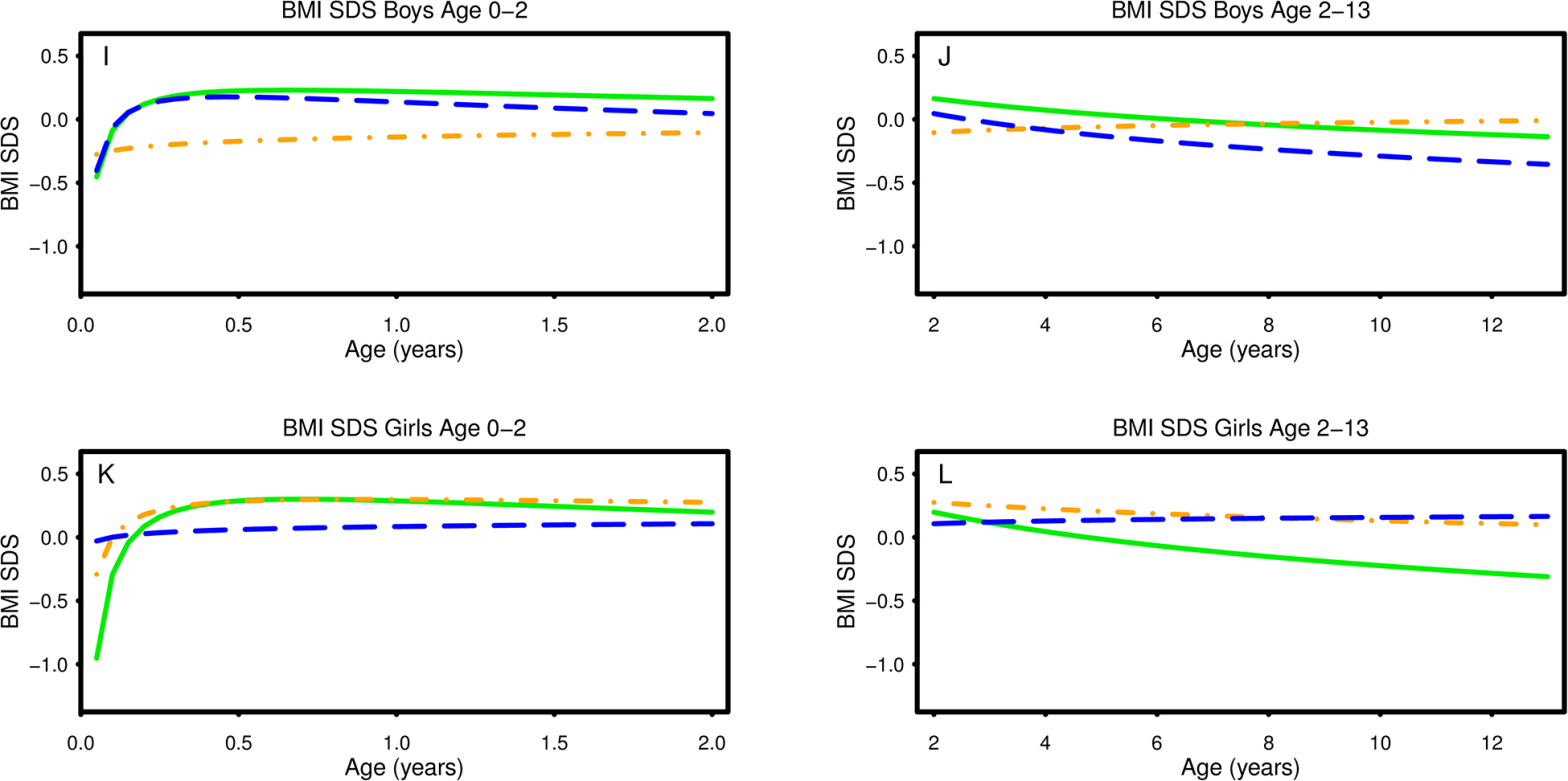
Continued from Fig 1. Plots of predicted BMI SDS (I-L) vs. age according to sex and severity of preeclampsia. Key to figures: Solid line = Unexposed, Dash-dot line = mild/moderate preeclampsia, Dashed line = Severe preeclampsia. Each figure represents the fractional polynomial (FP) with the best fit for each measure (X), i.e. FP (-0.5, 0) = -b_1_/√X + b_2_ln(X).

The growth curves for height, weight and BMI by sex and severity of PE as developed by fractional polynomial regression are shown in Figs 1 and 2. The corresponding regression models appear in Table 2.

**Table 2:** Multiple fractional polynomial regression of growth from birth to 13 years of age^a)^ using generalized estimating equations analyses in Norwegian children born in 1993–1995 according to mother’s preeclampsia status and interaction with age and sex.

Independent variables	Outcome variable								
	Length/height SDS *n* = 502			Weight SDS *n* = 502			BMI SDS *n* = 501		
	b	95% CI	*P*	b	95% CI	*P*	b	95% CI	*P*
**Preeclampsia (No = reference)**									
**Mild/Moderate**	-0.20	(-0.47, 0.08)		0.08	(-0.16, 0.33)		0.21	(-0.01, 0.44)	
**Severe**	**-0.54**	**(-0.99, -0.08)**		-0.19	(-0.59, 0.21)		0.14	(-0.24, 0.52)	
**Age**									
**FP1**	**0.21**	**(0.16, 0.26)**		**0.30**	**(0.15, 0.44)**		**-0.23**	**(-0.34, 0.13)**	
**FP2**	**-0.22**	**(-0.31, -0.12)**		**-1.32**	**(-1.87, -0.76)**		**-0.44**	**(-0.58, -0.30)**	
**Sex (male)**	**-0.19**	(-0.41, 0.04)		-0.07	(-0.27, 0.13)		0.05	(-0.14, 0.23)	
**Preeclampsia × Age**									
**Mild/Moderate × FP1**	-0.05	(-0.14, 0.04)		0.03	(-0.20, 0.26)		**0.13**	**(0.01, 0.26)**	
**Mild/Moderate × FP2**	0.12	(-0.04, 0.28)		0.34	(-0.57, 1.24)		**0.27**	**(0.09, 0.46)**	
**Severe × FP1**	-0.07	(-0.21, 0.07)		-0.05	(-0.39, 0.29)		**0.23**	**(0.01, 0.45)**	
**Severe × FP2**	**0.30**	**(0.03, 0.57)**		1.08	(-0.30, 2.44)		**0.47**	**(0.14, 0.80)**	
**Preeclampsia × sex**									
**Mild/Moderate × sex**	**0.43**	**(0.02, 0.84)**		-0.09	(-0.45, 0.27)		-0.32	(-0.65, 0.02)	
**Severe × sex**	-0.39	(-1.00, 0.22)		-0.38	(-0.91, 0.15)		-0.31	(-0.82, 0.20)	
**Sex × Age**									
**Sex × FP1**	-0.04	(-0.12, 0.04)		-0.12	(-0.33, 0.10)		0.10	(-0.05, 0.26)	
**Sex × FP2**	0.05	(-0.08, 0.19)		0.54	(-0.28, 1.35)		0.19	(-0.02, 0.39)	
**Sex × Preeclampsia × Age**			0.290			0.299			**0.020**
**Sex × Mild/Moderate × FP1**	0.13	(-0.01, 0.27)		-0.06	(-0.41, 0.30)		-0.001	(-0.23, 0.23)	
**Sex × Mild/Moderate × FP2**	-0.12	(-0.36, 0.12)		0.31	(-1.07, 1.68)		0.03	(-0.29, 0.35)	
**Sex × Severe × FP1**	0.01	(-0.17, 0.19)		0.04	(-0.35, 0.43)		-0.24	(-0.53, 0.07)	
**Sex × Severe × FP2**	-0.05	(-0.38, 0.29)		-0.66	(-2.27, 0.94)		**-0.52**	**(-0.97, -0.08)**	
**Birth order (firstborn)**	-0.03	(-0.20, 0.13)	0.698	-0.04	(-0.20, 0.11)	0.558	0.01	(-0.14, 0.17)	0.873
**Maternal age at delivery (years)**	0.01	(-0.01, 0.02)	0.486	-0.002	(-0.02, 0.01)	0.794	-0.002	(-0.02, 0.01)	0.819
**Maternal smoking (yes)**	**-0.24**	**(-0.41, -0.06)**	**0.007**	**-0.16**	**(-0.33, -0.002)**	**0.047**	0.03	(-0.13, 0.19)	0.719
**Maternal BMI (kg/m**^**2**^**)**	**0.02**	**(0.002, 0.04)**	**0.029**	**0.05**	**(0.03, 0.07)**	**<0.001**	**0.05**	**(0.03, 0.07)**	**<0.001**
**Maternal education**^b)^	-0.05	(-0.15, 0.06)	0.364	-0.05	(-0.14, 0.05)	0.356	-0.01	(-0.10, 0.09)	0.920
**Intercept**	-0.22	(-0.97, 0.52)		-0.76	(-1.44, -0.07)		-1.21	(-1.90, -0.52)	

*Abbreviations*: *n* = number of participants; CI = confidence interval; BMI = body mass index; SDS = standard deviation score; *P* = Wald chi-square test for interaction between preeclampsia and age; FP = fractional polynomial. FP1 (length/height SDS) = ln(X) + 0.8368401723; FP2 (length/height SDS) = X^3^ − 0.0812259488; where: X = (Age + 0.258516924726631)/10. FP1 (weight SDS) = ln(X) + 0.8404720262; FP2 (weight SDS) = √X−0.6568917665; where: X = (Age + 0.258516924726631)/10. FP1 (BMI) = 1/√X − 1.435656586; FP2 (BMI) = ln(X) + 0.7232445915; where: X = Age/10.

^a)^ Calculated from measurements at birth and the target ages of 3, 6 months, 1, 4, 10.8 (girls)/11.8 (boys) and 12.8 years;

^b)^ Maternal education at delivery: ≤9 years, 9–12 years, > 12 years.

For all the interactions involving preeclampsia, the category “no” preeclampsia is the reference.

The results of fully adjusted analysis of the interaction between sex, age and PE and the effect on height, weight and BMI appear in Table 2 and are described below.

### Length/Height SDS (Fig 1A–1D, Table 2)

*In utero* exposure to PE was associated with linear growth. Specifically, boys exposed to mild/moderate PE had an increased linear growth above 0.5 years compared to the unexposed. Boys exposed to severe PE had a decreased linear growth trajectory compared to the unexposed boys across all ages. As an example, boys exposed to severe PE were approximately 3 cm shorter than the unexposed boys at 2 years of age. Girls exposed to PE had a decreased linear growth trajectory until 12 years of age (non-significant difference between boys and girls; P < 0.290, Table 2). Girls exposed to severe PE were approximately 2 cm shorter than the unexposed girls at 2 years of age.

### Weight SDS (Fig 1E–1H, Table 2)

Weight SDS in children exposed to mild/moderate PE, and in girls exposed to severe PE, was lower than in the unexposed from birth through preschool age and higher thereafter, but the differences were maximally equivalent to 0.5 kg. Weight SDS in boys exposed to severe PE was lower than in the unexposed across all ages in childhood (non-significant difference between boys and girls; P = 0.299, Table 2). As an example, weight in boys exposed to severe PE was approximately on1 kg lower than in the unexposed boys at 2 years of age.

### BMI SDS (Fig 2I–2L, Table 2)

BMI SDS in boys exposed to mild/moderate PE was lower than in the unexposed from infancy through 7 years, e.g. at 6 months of age, BMI in boys exposed to mild/moderate PE was approximately 0.8 kg/m^2^ lower than in the unexposed boys. Boys exposed to severe PE experienced a lower BMI SDS than the unexposed boys across all ages, e.g. BMI in boys exposed to severe PE was approximately 0.1 kg/m^2^ lower than in the unexposed boys at 12 years of age. BMI SDS in girls exposed to mild/moderate PE was higher than in the unexposed from 1 year of age. Girls exposed to severe PE experienced a lower BMI SDS than the unexposed from infancy, but higher after 4 years of age, with the maximum difference of 1.5 kg/m^2^ at 12.8 years of age (significant difference between boys and girls; P = 0.020, Table 2).

### Other measurements

At both 10.8/11.8 (girls/boys) and 12.8 years, severe PE was positively associated with waist-to-height ratio SDS (Table 3). Finally, in multiple linear regression analyses, there were no associations between PE (both categories) and waist circumference SDS, triceps or scapular skinfold SDS at any age (S1, S2 and S3 Tables).

**Table 3:** Multiple linear regression analysis of waist-to-height ratio SDS in 586 Norwegian children born in 1993–1995 according to mother’s preeclampsia status.

Independent variables	10.8/11.8 years^a)^, *n* = 519			12.8 years, *n* = 390		
	b	95% CI	*F*-test *P*^b)^	b	95% CI	*F*-test *P*^b)^
Intercept	-1.57	(-2.15, -0.98)	< 0.001	-0.93	(-1.53, -0.33)	0.002
Preeclampsia			**0.011**			**0.019**
None	0.00	Reference		0.00	Reference	
Mild/moderate	0.16	(-0.06, 0.38)		-0.03	(-0.25, 0.20)	
Severe	**0.47**	**(0.15, 0.79)**		**0.44**	**(0.12, 0.75)**	
Sex (male)	-0.07	(-0.26, 0.11)	0.435	-0.15	(-0.35, 0.04)	0.124
Maternal BMI (kg/m^2^)	**0.07**	**(0.04, 0.09)**	**< 0.001**	**0.06**	**(0.03, 0.08)**	**< 0.001**
Maternal smoking (yes)	**0.25**	**(0.03, 0.47)**	**0.024**	0.21	(-0.02, 0.45)	0.072

*Abbreviations*: *n* = number of participants; CI = Confidence interval; SDS = Standard deviation scores; *F*-test *P* refers to exposure only; BMI = body mass index (kg/m^2^).

Bold numbers indicate statistical significance

^a)^ 10.8 years for girls, 11.8 years for boys

^b)^ After backward stepwise selection from fully adjusted model.

## Discussion

In the present study of children exposed and unexposed to PE *in utero*, absolute values for and trajectories in length/height, weight and BMI from birth to late childhood differed by sex and severity of PE. Mild/moderate PE was in general positively associated with the development of length/height in boys, and with weight and BMI for both sexes. Severe PE was in general negatively associated with the development of length/height, weight and BMI except in girls, where severe PE was positively associated with weight and BMI after preschool ages. Severe PE was also associated with a larger waist-to-height ratio in late childhood in both sexes.

Apart from the Stavanger Study [3, 10], three earlier studies examined the association between PE and childhood growth. In all of those three studies, PE was examined as one entity rather than by severity. First, in a Norwegian study of 4096 girls aged 13–19 years PE was positively associated with BMI, and our results are in accord [12]. Second, in a large cohort of Israeli adolescents, exposure to PE (n = 428) was positively associated with weight and BMI at 17 years of age in boys but not girls [22]. This corresponds to our findings in boys exposed to mild/moderate preeclampsia, but not severe preeclampsia. Third, in a study of three cohorts with a total of 4622 children, those exposed to PE had low birthweight, but catch-up growth postnatally [9]. This corresponds to our results in children exposed to mild/moderate PE, and girls, but not boys, exposed to severe PE. The discrepancies across studies may be due to different designs namely different ages at assessment and because in the other three studies [9, 12, 22] PE was not differentiated by severity.

To our knowledge, no previous publications exist on the associations between PE exposure and linear growth. In our study, boys exposed to mild/moderate PE had a recumbent length at birth similar to that of unexposed, but exceeded linear growth during preschool age, while those exposed to severe PE had a decreased linear growth compared to unexposed at all ages. PE had less impact on linear growth in girls, however both mild/moderate and severe PE was negatively associated with linear growth in girls. The decreased linear growth after exposure to severe PE is in contrast with studies on fetal growth retardation where postnatal catch-up in linear growth is reported [23, 24]. However, studies on very premature children born small for gestational age have reported that they may be less likely to have catch-up in linear growth, or only after 6 years of age [25]. IGF-I is one of the most important regulators of postnatal growth and is known to be lower in placental tissues and cord blood in women with severe, but not mild, PE [26, 27]. Therefore, poor linear growth in children exposed to severe PE might be due to effects on the growth hormone- IGF-1 axis [28–30], an effect that might be mediated through inflammation or perhaps the result of fetal programming. PE is characterized by inflammation and the cytokines of pregnancy could correlate with those in offspring until the age of 1 year [31, 32]. Furthermore, a pro-inflammatory status might induce apoptosis of the growth plate cartilage both prenatally and during infancy [33].

Weight and BMI were lower in children exposed to mild/moderate PE, and girls exposed to severe PE, but weight and BMI in these children were higher from school age and onwards when compared to unexposed children. This effect is similar to what is found in other children born small for gestational age [25, 34] and in prematurely born children [35]. Prenatal starvation is associated with epigenetic changes that could persist throughout life, causing a tendency for energy conservation and thus overweight [36, 37]. Children with catch-up growth postnatally have better insulin sensitivity than other children, so these children will have a favorable linear growth and weight development [38]. However, most children born small for gestational age catch up in weight and length before 12 months of age [25], but this was not the case in our study subjects who experienced catch-up at a later age. This difference suggests that PE affects weight and BMI independently of small for gestational age status. Boys exposed to severe PE had lower weight and BMI during infancy and childhood when compared to the unexposed. These results are different from children born prematurely or small for gestational age. The effects of severe PE on weight trajectories in boys could be influenced by some of the same mechanisms as those for linear growth, like low levels of IGF-1, mediated through prematurity and inflammation [39].

Children exposed to severe PE had a higher waist-to-height ratio than the unexposed children in late childhood. Similar results have been found in children born small for gestational age, who continue to gain excess body fat even after catch-up in weight [25]. A high waist-to-height ratio is a known risk factor for insulin resistance and metabolic syndrome [34]. As severe PE is characterized by inflammation [40, 41], and studies indicate an association between inflammatory disorders in adults and metabolic syndrome [42] one can speculate that exposure to severe PE might indirectly and via inflammation be a risk factor for metabolic syndrome.

The current study shows different effects of mild/moderate and severe PE on childhood growth. This might be explained by different pathophysiology of the two conditions. Mild/moderate PE more often appears late whereas severe PE appears early in pregnancy. Early and late-onset PE differ by maternal age, pre-pregnancy BMI, maternal cardiac output, vascular resistance and endothelial damage [15, 41, 43]. Further, only early onset PE is associated with fetal growth restriction, due to incomplete invasion of trophoblast into the maternal spiral arteries, and changes in blood flow in umbilical arteries [4, 41]. Mild/moderate PE is more common in overweight mothers with metabolic syndrome, while severe PE is more common in normal weight women, but possibly characterized by more inflammation; thereby reflecting different maternal phenotypes by severity of PE with implications for child growth [15, 40].

There were differences between the sexes regarding the effect of PE on length/height and weight, and significant differences between the sexes regarding the effect of PE on BMI. While PE exposure in girls had some negative effects on linear growth, in boys mild/moderate PE exposure was generally positively associated, and severe PE negatively associated with linear growth. PE was generally positively associated with weight and BMI in boys and girls, while in boys only, severe PE was generally negatively associated with weight and BMI. Although there are inconsistencies in the literature regarding sex differences in growth after exposure to PE [10, 12, 22], boys are generally more prone to neonatal complications [44]; whether born to term and of adequate birthweight, small for gestational age [45] and extreme prematurity affects boys more severely than it does to girls [46].

### Statistical considerations

We did not adjust for gestational age or birthweight in the analyses because they are intermediary variables on the causal pathway from PE to growth in childhood, and adjusting for them could attenuate the association between PE and growth. Further, as gestational age and birthweight may be influenced by unknown factors, adjusting for them could give rise to spurious associations between PE and growth. Therefore, modeling conditional growth (and thereby correcting for the regression to the mean) was not feasible.

Other strategies to compare growth between the three groups unexposed, exposed to mild/moderate and exposed to severe PE could have been pursued if we wanted to separate from the effects of gestational age and child’s birth weight. Stratification is a statistically inferior method of adjusting, and as explained, adjustment for these variables cannot be done. Further, stratification would lead to a loss of power if used.

For the same reasons, we did not adjust for pubertal status. Some effects of PE could be indirect and mediated through prematurity and being born small for gestational age, but the distinct differences in growth between children exposed to PE from those who are otherwise born small for gestational age indicates that PE has effects on growth other than through pathways related to gestational age or birthweight.

The extremes of Figs 1 and 2 should be interpreted with caution. The mathematical model attempting to predict the best curve might be influenced by very few observations of length SDS and weight SDS before age 0.0 years, particularly in the group of mild/moderate PE and in the unexposed group.

### Strengths and limitations

An advantage of the study population is its homogeneity of socio-economic status and ethnicity. Furthermore, the measurements of height and weight were sampled repeatedly during childhood, enabling the possibility of calculating a predictive model of growth, i.e., the length/height, weight or BMI of a child at a given age, by sex or PE exposure status.

The study also has some limitations. The original matching on maternal age and birth date in the analyses was compromised due to more missing participants in the unexposed than the exposed group, which may be a source of bias. However, we adjusted for maternal age in the statistical analyses to avoid confounding bias. We described above why the analyses could not be adjusted for gestational age or birthweight; therefore, the study might be biased. A matching of participants on gestational age and birthweight could have reduced bias. The anthropometric measurements from the well-baby clinics were not measured by the research team; however, routine measurements at well-baby clinics are standardized. Repeated measurements of waist circumference (a more accurate measurement of adiposity) and skinfolds were not performed at well-baby clinics [17]. Furthermore, we do not have consecutive anthropometric measurements across ages in childhood. There was a considerable attrition at the second follow-up of around 50%, which may be a source of selection bias. However, there were no known perinatal differences between those who assented to the first follow-up and those who did not assent, and most baseline characteristics between those who participated in the first and the second follow-ups did not differ. The average BMI of girls who participated in the first but not the second follow-up was higher than the BMI of girls who participated in both follow-ups. However, there is no reason to assume that the girls with the higher BMI who were lost to the second follow-up were overrepresented in any particular exposure group. Therefore, the association between PE and growth would not be expected to be biased by the change in BMI of the girls from the first to the second follow-up.

## Conclusions

From birth to adolescence, linear growth, weight and BMI trajectories differed among the PE exposed children by severity of PE, age and sex compared with the unexposed. In general, PE exposure was negatively associated with linear growth, while in girls positive associations with weight and BMI were observed. The trajectories might differ from those found in studies of children born small for gestational age. The present results underline that fetal life is a particularly sensitive period regarding growth, supporting the hypothesis that an adverse intrauterine environment may affect postnatal development, anthropometry throughout childhood and probably also the metabolic phenotype. Our results may have implications for targeted approaches for healthy growth and development.

## Supporting information

S1 Fig Directed acyclic graph of the association between preeclampsia and growth with confounders. Green with arrowhead = exposure, blue with black bar = outcome, red = ancestor of exposure and outcome, blue = ancestor of outcome. Green arrows = causal path, red arrows = biasing path.
(PDF)

S1 Table Multiple linear regression analyses of waist circumference SDS at 10.8/11.8 and 12.8 years of age in 593 children according to mother’s preeclampsia status. (DOCX)

S2 Table Multiple linear regression analyses of triceps skinfold SDS at 10.8/11.8 and 12.8 years of age in 589 children according to mother’s preeclampsia status. (DOCX)

S3 Table Multiple linear regression analyses of waist circumference SDS at 12.8 years of age in 487 children according to mother’s preeclampsia status. (DOCX)

S1 Statistics File SPSS dataset. (SAV)

S2 Statistics File STATA dataset. (DTA)

S3 Statistics File Text document with script for R. (R)
