# Supplementary figures and images for: Exposure to preeclampsia in utero affects growth from birth to late childhood dependent on child’s sex and severity of exposure: Follow-up of a nested case-control study
Source: PLoS One. 2017 May 9;12(5):e0176627. doi: 10.1371/journal.pone.0176627 (PMC5423584; doi:10.1371/journal.pone.0176627)

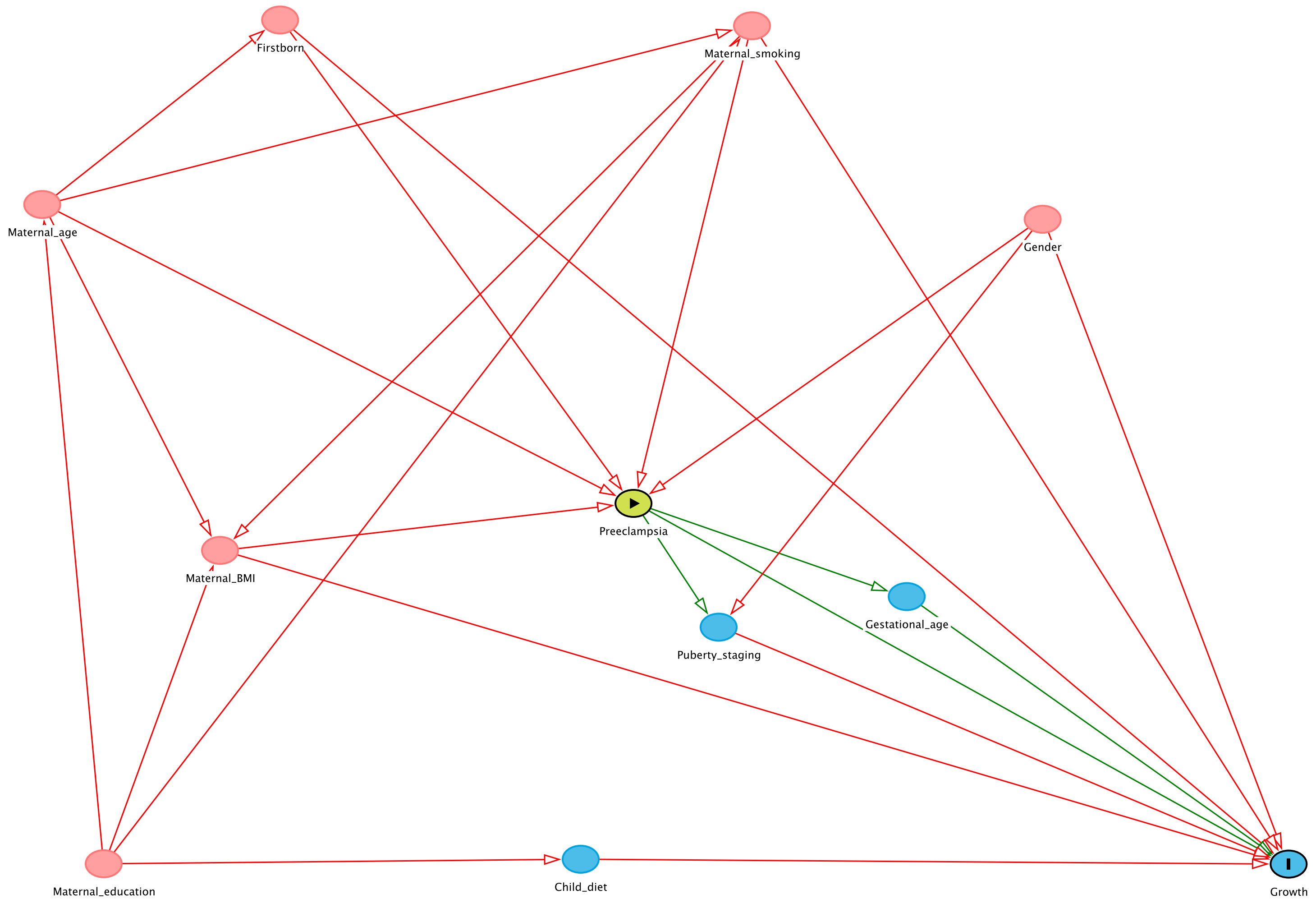

Supplement: S1 Fig — Green with arrowhead = exposure, blue with black bar = outcome, red = ancestor of exposure and outcome, blue = ancestor of outcome. Green arrows = causal path, red arrows = biasing path. (PDF) [file pone.0176627.s001.pdf]
