# Supplementary material for: Exposure to preeclampsia in utero affects growth from birth to late childhood dependent on child’s sex and severity of exposure: Follow-up of a nested case-control study
Source: PLoS One. 2017 May 9;12(5):e0176627. doi: 10.1371/journal.pone.0176627 (PMC5423584; doi:10.1371/journal.pone.0176627)
Supplement: S1 Table — (DOCX) [file pone.0176627.s002.docx]

| **S1 table. Multiple linear regression analyses of waist circumference SDS at 10.8/11.8 and 12.8 years of age in 593 children according to mother’s preeclampsia status** | | | | | | |
| --- | --- | --- | --- | --- | --- | --- |
|  | **10.8/11.8 years^a)^, *n =* 519** | | | **12.8 years, *n =* 390** | | |
| **Independent variables** | **b** | **95 % CI** | ***F*-test *P*** | **b** | **95 % CI** | ***F*-test *P*** |
| Intercept | -1.71 | (-2.29, -1.13) | < 0.001 | -1.09 | (-1.70, 0.48) | 0.001 |
| Preeclampsia |  |  | 0.098 |  |  | 0.344 |
| None | 0.00 | Reference |  | 0.00 | Reference |  |
| Mild/moderate | 0.18 | (-0.04, 0.40) |  | 0.04 | (-0.19, 0.27) |  |
| Severe | 0.27 | (-0.14, 0.53) |  | 0.24 | (-0.08, 0.56) |  |
| Sex (male) | -0.09 | (-0.28, 0.10) | 0.352 | -0.19 | (-0.39, 0.01) | 0.056 |
| Maternal BMI (kg/m^2^) | 0.08 | (0.05, 0.10) | < 0.001 | 0.06 | (0.04, 0.09) | < 0.001 |
| Maternal smoking (yes) | 0.11 | (-0.10, 0.33) | 0.301 | 0.12 | (-0.12, 0.36) | 0.331 |
