# Supplementary material for: Exposure to preeclampsia in utero affects growth from birth to late childhood dependent on child’s sex and severity of exposure: Follow-up of a nested case-control study
Source: PLoS One. 2017 May 9;12(5):e0176627. doi: 10.1371/journal.pone.0176627 (PMC5423584; doi:10.1371/journal.pone.0176627)
Supplement: S2 Table — (DOCX) [file pone.0176627.s003.docx]

| **S2 table. Multiple linear regression analyses of triceps skinfold SDS at 10.8/11.8 and 12.8 years of age in 589 children according to mother’s preeclampsia status** | | | | | | |
| --- | --- | --- | --- | --- | --- | --- |
|  | **10.8/11.8 years^a)^, *n =* 515** | | | **12.8 years, *n =* 389** | | |
| **Independent variables** | **b** | **95 % CI** | ***F*-test *P*** | **b** | **95 % CI** | ***F*-test *P*** |
| Intercept | -1.72 | (-2.31, -1.13) | < 0.001 | -1.31 | (-1.97, -0.66) | < 0.001 |
| Preeclampsia |  |  | 0.388 |  |  | 0.903 |
| None | 0.00 | Reference |  | 0.00 | Reference |  |
| Mild/moderate | 0.15 | (-0.07, 0.37) |  | -0.03 | (-0.27, 0.22) |  |
| Severe | 0.03 | (-0.29, 0.34) |  | 0.06 | (-0.29, 0.41) |  |
| Sex (male) | 0.20 | (0.02, 0.39) | 0.031 | 0.02 | (-0.19, 0.24) | 0.835 |
| Maternal BMI (kg/m^2^) | 0.06 | (0.03, 0.08) | < 0.001 | 0.05 | (0.02, 0.08) | < 0.001 |
| Maternal smoking (yes) | 0.06 | (-0.15, 0.28) | 0.570 | -0.10 | (-0.36, 0.16) | 0.437 |
