# Supplementary material for: Exposure to preeclampsia in utero affects growth from birth to late childhood dependent on child’s sex and severity of exposure: Follow-up of a nested case-control study
Source: PLoS One. 2017 May 9;12(5):e0176627. doi: 10.1371/journal.pone.0176627 (PMC5423584; doi:10.1371/journal.pone.0176627)
Supplement: S3 Table — (DOCX) [file pone.0176627.s004.docx]

| **S3 table. Multiple linear regression analyses of waist circumference SDS at 12.8 years of age in 487 children according to mother’s preeclampsia status** | | | |
| --- | --- | --- | --- |
|  | **12.8 years, *n =* 380** | | |
| **Independent variables** | **b** | **95 % CI** | ***F*-test *P*** |
| Intercept | -2.13 | (-2.74, -1.52) | < 0.001 |
| Preeclampsia |  |  | 0.384 |
| None | 0.00 | Reference |  |
| Mild/moderate | 0.15 | (-0.07, 0.38) |  |
| Severe | 0.10 | (-0.22, 0.43) |  |
| Sex (male) | 0.12 | (-0.08, 0.31) | 0.241 |
| Maternal BMI (kg/m^2^) | 0.07 | (0.04, 0.10) | < 0.001 |
| Maternal smoking (yes) | 0.13 | (-0.11, 0.37) | 0.284 |
